# Effects of Imprinting Pressure on the Damage of Flexible Composite Mould and Pattern Quality during UV Nanoimprinting

**DOI:** 10.3390/mi10100706

**Published:** 2019-10-17

**Authors:** Xu Zheng, Qing Wang, Wenquan Du

**Affiliations:** Institute of NanoEngineering, College of Civil Engineering and Architecture, Shandong University of Science and Technology, Qingdao 266590, China; zhengxu081@163.com (X.Z.); 13021662108@163.com (W.D.)

**Keywords:** composite mould, pattern quality, imprinting pressure, UV nanoimprint

## Abstract

Imprinting pressure is the significant factor for composite mould durability and pattern quality during UV nanoimprinting on complex surfaces. To solve these problems, the effects of imprinting pressure on the damage of flexible composite mould and pattern quality-encountering particles were investigated through experiment and simulation. It was found that increasing the pressure could improve the pattern quality, but it will damage the mould and reduce the durability. Moreover, too small pressure could lead to serious pattern defects. Therefore, the imprint pressure of 30 kPa was suitable for use in the imprinting process from the viewpoints of protecting the mould and reducing pattern defects. These findings will be useful for improving the pattern quality and mould durability.

## 1. Introduction

The preparation of high quality and precision moulds is the core of nanoimprint lithography (NIL), and the hard mould and the soft mould are the widest applications at present [1,2]. In the case of the hard mould used for NIL under high temperature and pressure, it is not only easy to damage the mould, but also easy to cause pattern defects in the demoulding process [3,4,5,6]. For the soft mould, it can well meet the requirements in term of high throughput, low cost and the duplicate capability on curved surfaces [7,8,9,10,11]. However, the low elastic modulus and the poor durability limit the achievable resolution and lifetime [12,13,14,15]. The composite mould can be conformably contacted with a complex substrate compared to the hard mould, and its high Young’s modulus ensures a higher pattern resolution compared to the soft mould. The composite mould extends the application range of nanoimprint from planar substrates to high-curvature surfaces or complex non-planar surfaces [16]. Therefore, the composite mould has a good application in the field of fabricating artificial compound eyes, hemispherical electronic eye cameras, photovoltaic devices, image sensor array, micro scale components, fiber optic sensor and other curved surface devices [17,18,19,20]. 

The flexible composite mould consists of a structural top layer and a flexible supporting backplane. Researchers successfully prepared high-resolution and curved patterns using hard- polydimethylsiloxane (H-PDMS)/PDMS, H-PDMS/acrylate and polyimide/Ormo composite moulds [21,22,23]. Although composite moulds can achieve higher resolution patterns on complex surfaces, defects such as interface separation and fracture of the support layer limit the application [24]. Furthermore, the damage of structural layer is caused by uneven substrates, and in particular, dust particles in the air cannot be ignored. The flexibility of the composite mould contributes to the replication of the pattern in the presence of particulate conditions, but it still causes damage to the pattern quality and mould durability. In this case, the setting of the imprinting pressure is a key factor for reducing the damage of the composite mould and improving the quality of the pattern. The objective of this research is to explore the effects of imprinting pressure on the damage of flexible composite mould and pattern quality encountering particle during UV-NIL.

In this paper, two major factors causing mould and pattern destruction in the imprinting process, i.e., the imprinting pressure and particles size were investigated through experiment. The maximum stress of composite mould during the imprting process was studied by finite element method (FEM) simulation of these two factors. The simulation results revealed the variation of the maximum stress and pattern damage range with various pressures and particles sizes. The simulation model and numerical results were useful for improving the pattern quality and mould durability

## 2. Methods and Modeling

### 2.1. Experiment

**Figure 1:**
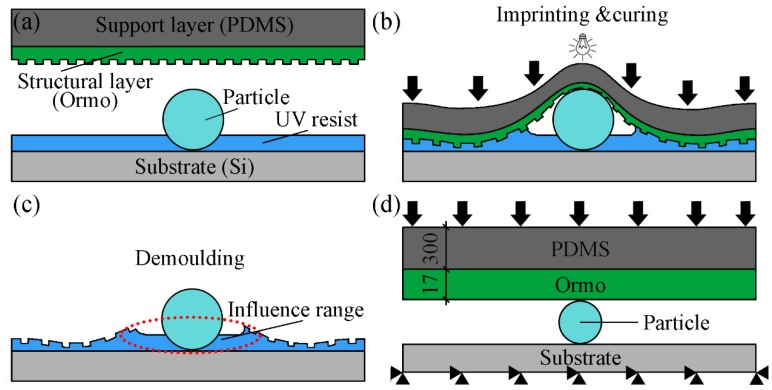
(**a**–**c**) schematic diagram of composite mould imprinting process encountered particles, (**d**) schematic diagram of geometric model and boundary condition in simulation.

UV-NIL was used to explore the effects of imprinting pressures and particles on the pattern quality. As shown in Figure 1a, the UV resist (AZ5214E, AZ Electronic Materials, Somerville, USA) with the thickness of 1.4 μm was spin-coated on the surface of silicon wafer at 4000 rpm for 40 s. Then, the 4 μm particles were placed on the surface of UV resist. The PDMS/Ormo composite mould with a period of 5 μm was employed during the UV-NIL process. Based on the neutral layer theory, the composite mould with 300 μm support layer (PDMS) and 17 μm structural layer (Ormo) were calculated for the experiments and simulation to reduce the interfacial damage of the mould during the imprinting process [25]. As shown in Figure 1b, the UV resist was imprinted and cured under the pressure of 50 and 200 kPa for 10 min in the nanoimprinting equipment (NIL-150, Imprint Nano, Nanjing, China), respectively. The composite mould was separated from the patterned resist as shown in Figure 1c. It is found that the quality of the pattern will be greatly affected if particles are mixed during the imprinting process.

### 2.2. Numerical Simulation

To explore factors affecting the quality of the pattern obtained after demoulding, the imprinting process with different pressures and particles were investigated by the finite element method (FEM) using ANSYS software (ANSYS 15.0, ANSYS, Pittsburgh, USA) [26,27]. As shown in Figure 1d, the geometrical model was established, which was consist of the support layer (PDMS, 300 μm) and structural layer (Ormo, 17 μm). The size of the PDMS in the model is the same as the thickness of the spin coating in experiment. In the simulation, the model is assumed to be uniform. Moreover, the PDMS and Ormo were defined as the rubber elastic material, and the particle and substrate were defined as rigid materials. The Mooney-Rivlin model was used to describe the PDMS and Ormo layers [28,29]. The Young’s moduli and Poisson ratios are applied to demonstrate its mechanical properties. In all simulations, the Young’s moduli of the PDMS layer and Ormo layer were 2 and 650 MPa, respectively. The Poisson ratios of the PDMS layer and Ormo layer were 0.45 and 0.22, respectively. 

Regarding the boundary conditions, the horizontal and vertical directions of the particle and substrate were fixed. The mould was a free boundary condition that could move freely in both horizontal and vertical directions. In the simulation, the element PLANE 42 was used to represent the particle and substrate, and the element PLANE 182 was used to represent the PDMS/Ormo composite mould. The contact element of CONTA 171 and TARGE169 ware applied for the interface between the PDMS/Ormo composite mould. To study the influences of the imprint pressure and particle radius on pattern quality, varied parameters were simulated. During the imprinting process, the uniform load was set on surface of composite mould from 10 to 100 kPa. The particle with radius of 2, 4, 6, and 8 μm were investigated.

## 3. Results and Discussions

### 3.1. Morphology of the Fabricated Grating Pattern

**Figure 2:**
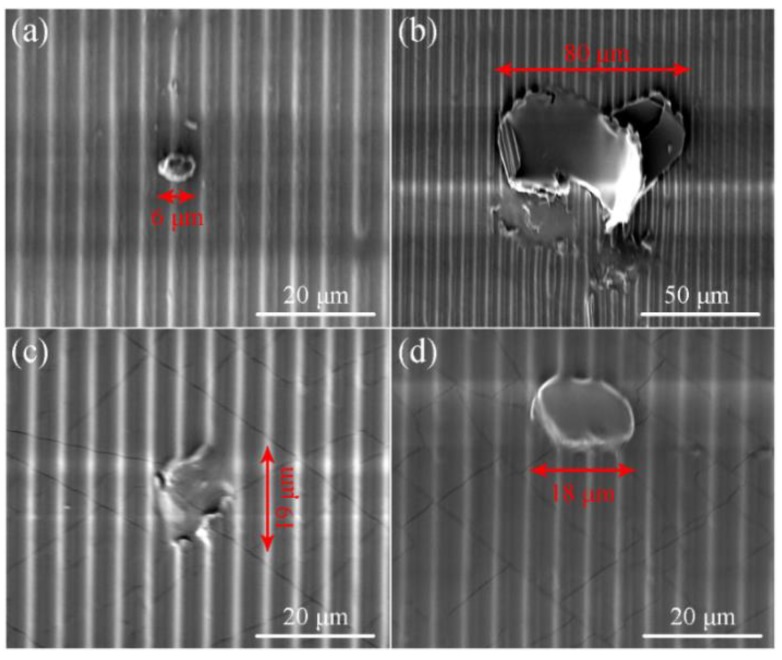
(**a**) Scanning electron microscopy (SEM) images of the mould with adheredparticle. SEM images of the grating patterns under the imprint pressure of (**b**) 50, (**c**) 150 and (**d**) 200 kPa when the particles radius of 4 μm.

Figure 2 shows the scanning electron microscopy (SEM) images of the mould and imprinted grating patterns with particles. As shown in Figure 2a, a 6 μm diameter particle is adhered to the mould surface with a period of 5 μm. Figure 2b shows that the grating pattern was damaged in a large area with the influence range of 80 μm under a pressure of 50 kPa when the particles radius of 4 μm. The main defects of the pattern are pattern loss and edge damage. The cause of this form of damage may be due to the low imprinting pressure. As shown in Figure 2c,d, the influence range decreases to 19 and 18 μm when the pressure changes to 150 and 200 kPa. However, excessive pressure will not only cause cracks on the surface of the pattern but also cause damage to the mould. This result suggests that the imprinting pressure is an important factor affecting the quality of the pattern and the protection of the mould. Therefore, this influence factor was explored through imprinting on various particle sizes using FEM simulations.

### 3.2. Von Mises Stress Distribution and Defects

**Figure 3:**
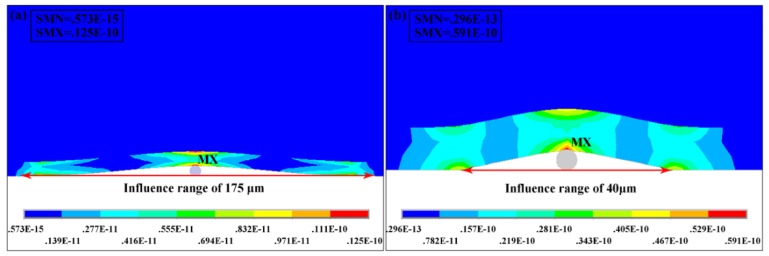
Von Mises stress distribution and influence range of composite mould for (**a**) particle radius of 6 μm under the pressure of 20 kPa and (**b**) particle radius of 2 μm under the pressure of 80 kPa.

**Figure 4:**
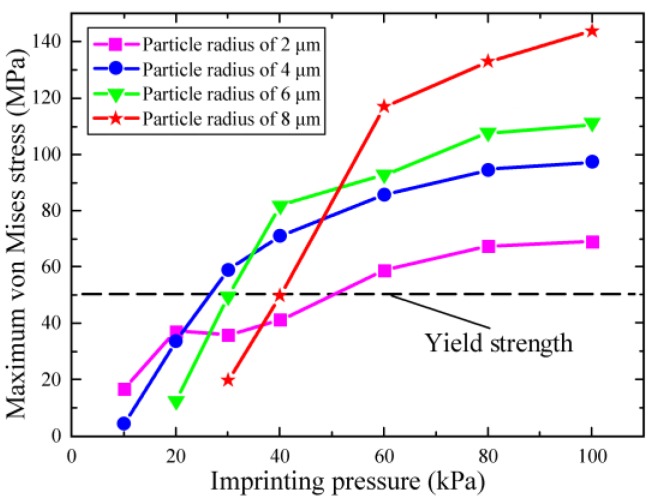
The maximum von Mises stress of composite mould for various pressures on the particles radius from 2 to 8 μm.

Figure 3a shows the von Mises stress distribution in composite mould with a pressure of 20 kPa during the impringting process when the particle size is 6 μm. The stress is concentrated at the center of the structural layer (MX mark in Figure 4). The maximum stress value is 12 MPa which is less than yield strength (50 MPa) of Ormo-structural layer. Therefore, the mould will only produce elastic deformation during the process of imprinting. However, the particle has a large influence range of 175 μm which cause great defects in the pattern. Figure 3b shows the von Mises stress distribution with a pressure of 80 kPa when the particle size is 2 μm. The maximum stress at the center of the structural layer is 59 MPa which is greater than the yield strength of 50 MPa. Although the influence range of the pattern is only 40 μm, the maximum stress will damage the mould.

### 3.3. Effects of Imprinting Pressure on Composite Mould

To explore the effects on the maximum stress of composite mould and influence range of pattern, various particle sizes were simulated under the imprint pressures from 10 to 100 kPa by the FEM. Figure 4 illustrates the maximum von Mises stress at the center of the composite mould, for particle radius ranging from 2 to 8 μm. It can be found that the maximum stress of the mould increases with increasing imprint pressure under the same particle size. The dashed line indicates the yield strength of the structural layer. The maximum stress is not obviously affected by particle size when the imprint pressure is lower than 40 kPa. However, the maximum stress of the structure layer increases significantly when the imprint pressure is greater than 40 kPa. The maximum stress value reaches 147 MPa with 100 kPa pressure under particle radius of 8 μm. The plastic deformation can cause the mould to be damaged when the maximum stress is greater than the yield strength. Therefore, the maximum stress of the mould is lower than 50 MPa except for the particle radius is 4 μm when the imprint pressure is 30 kPa. This indicates that the composite mould only exhibits elastic deformation during the imprinting process when the pressure is less than 30 kPa.

### 3.4. Effects of Imprinting Pressure on Pattern Quality

**Figure 5:**
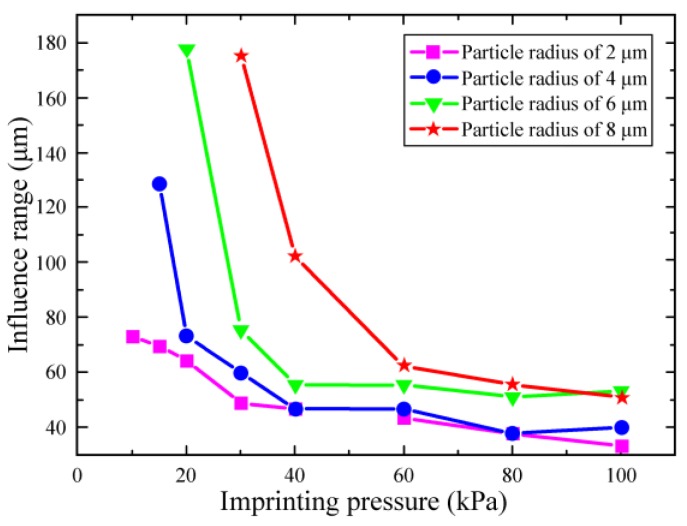
The influence range of fabricated patterns for various pressures on the particles radius from 2 to 8 μm.

The imprinting pressure is also an important factor for the defects forming in the experimentally prepared patterns. Figure 5 shows the influence range of particle on the pattern under different imprint pressures. It can be found that the influence range decreases with the increasing of pressure, indicating the pressure can effectively reduce the effect of particles on pattern quality. Moreover, the contact area between structural layer and the substrate increases with an increase of pressure. As shown in Figure 5, when the pressure is 30 kPa, the influence range rapidly reduces to an acceptable range with the particle radius from 2 to 6 μm. Furthermore, increasing pressure has little effect on the influence range when the pressure is greater than 40 kPa. Moreover, according to the discussion above, the mould will be damaged when the pressure is greater than 30 kPa. Therefore, the imprinting pressure of 30 kPa is suitable for use in the imprinting process from the viewpoints of protecting mould and reducing patterns defects.

## 4. Conclusions

A defective grating pattern was fabricated by UV-NIL on the UV resist with particles on the surface to simulate the dust encountered in the imprint experiment. It was found that excessive pressure could cause the mould to be damaged; too small pressure could lead to serious pattern defects. Therefore, imprinting pressure was an important factor affecting the quality of the pattern and the protection of the mould which were explored through imprinting on various particle sizes using FEM simulations. For a constant particle size, the maximum stress of the mould increased with the imprint pressure increased. When the imprint pressure exceeded 40 kPa, the maximum stress at the center of the structural layer was greater than the yield strength of 50 MPa which caused the mould to be damaged with plastic deformation. However, the increasing pressure could effectively reduce the effect of particles on pattern quality. It was found that the influence range of particles rapidly reduced to an acceptable range with a particle radius from 2 to 6 μm when the pressure was 30 kPa. Moreover, increasing pressure had little effect on the influence range when the pressure was greater than 40 kPa. Therefore, the imprint pressure of 30 kPa was suitable for use in the imprinting process from the viewpoints of protecting mould and reducing patterns defects. These findings will be useful for improving the pattern quality and mould durability.
